# Resveratrol Attenuates the Mitochondrial RNA-Mediated Cellular Response to Immunogenic Stress

**DOI:** 10.3390/ijms24087403

**Published:** 2023-04-17

**Authors:** Jimin Yoon, Doyeong Ku, Minseok Lee, Namseok Lee, Sung Gap Im, Yoosik Kim

**Affiliations:** 1 Department of Chemical and Biomolecular Engineering, Korea Advanced Institute of Science and Technology (KAIST), Daejeon 34141, Republic of Korea; jimyoon@kaist.ac.kr (J.Y.); jkn1996@kaist.ac.kr (D.K.); minseoklee@cornell.edu (M.L.); udklns716@kaist.ac.kr (N.L.); sgim@kaist.ac.kr (S.G.I.); 2 KAIST Institute for NanoCentury (KINC), Daejeon 34141, Republic of Korea; 3 KAIST Institute for Health Science and Technology (KIHST), Daejeon 34141, Republic of Korea; 4 KAIST Institute for BioCentury (KIB), Daejeon 34141, Republic of Korea; 5 BioProcess Engineering Research Center and BioInformatics Research Center, KAIST, Daejeon 34141, Republic of Korea

**Keywords:** resveratrol, Sjögren’s syndrome, innate immunity, immunogenic stress, dsRNA stress, oligomycin A, tunicamycin, mitochondrial double-stranded RNAs

## Abstract

Human mitochondria contain a circular genome that encodes 13 subunits of the oxidative phosphorylation system. In addition to their role as powerhouses of the cells, mitochondria are also involved in innate immunity as the mitochondrial genome generates long double-stranded RNAs (dsRNAs) that can activate the dsRNA-sensing pattern recognition receptors. Recent evidence shows that these mitochondrial dsRNAs (mt-dsRNAs) are closely associated with the pathogenesis of human diseases that accompany inflammation and aberrant immune activation, such as Huntington’s disease, osteoarthritis, and autoimmune Sjögren’s syndrome. Yet, small chemicals that can protect cells from a mt-dsRNA-mediated immune response remain largely unexplored. Here, we investigate the potential of resveratrol (RES), a plant-derived polyphenol with antioxidant properties, on suppressing mt-dsRNA-mediated immune activation. We show that RES can revert the downstream response to immunogenic stressors that elevate mitochondrial RNA expressions, such as stimulation by exogenous dsRNAs or inhibition of ATP synthase. Through high-throughput sequencing, we find that RES can regulate mt-dsRNA expression, interferon response, and other cellular responses induced by these stressors. Notably, RES treatment fails to counter the effect of an endoplasmic reticulum stressor that does not affect the expression of mitochondrial RNAs. Overall, our study demonstrates the potential usage of RES to alleviate the mt-dsRNA-mediated immunogenic stress response.

## 1. Introduction

Human mitochondria contain a compact circular genome of 16,569 DNA base pairs that encodes 13 protein-coding genes as well as 2 rRNAs and 22 tRNAs [1]. The proteins encoded by the mitochondrial genome are all subunits of the complexes participating in the oxidative phosphorylation system to produce cellular ATPs [2]. In addition to their traditional role as the powerhouses of the cells, one emerging function of mitochondria is the regulation of innate immunity [3]. The outer membrane of mitochondria hosts mitochondrial antiviral-signaling proteins (MAVS) that act as essential downstream factors for melanoma differentiation-associated protein 5 (MDA5) and retinoic acid-inducible gene-I (RIG-I) to drive innate immunity against double-stranded RNAs (dsRNAs) [4]. In addition, mitochondrial DNA (mtDNA) can also trigger innate immunity as its unique methylation pattern makes it appear to be of foreign origin [5]. In this context, the mtDNA acts as an agonist for Toll-like receptor 9 (TLR9) and induces a type I interferon (IFN) response via the cytosolic cyclic GMP-Amp synthase (cGAS)-stimulator of the interferon genes (STING) pathway [6,7].

Recently, mitochondrial RNAs (mtRNAs) have been receiving increasing attention as potent inducers of innate immunity [8,9,10]. Due to the bidirectional transcription of the circular genome, the mtDNA produces long complementary RNAs that can bind to each other to form intermolecular dsRNAs [11]. When released to the cytosol, these mitochondrial dsRNAs (mt-dsRNAs) are recognized by MDA5 and protein kinase R (PKR) to trigger the type I IFN response and initiate apoptotic programs [8,9,10,12]. The cytosolic efflux of mt-dsRNAs occurs through the Bax/Bak pore either due to mutations in the polyribonucleotide nucleotidyltransferase 1 (PNPT1) gene or by stressors that disturb the mitochondrial membrane potential [8,9,10,12]. Notably, recent studies reported a close association between the cytosolic release of mt-dsRNAs in the pathogenesis of human inflammatory diseases and aberrant immune activation. For instance, under alcohol-induced liver injury, mt-dsRNAs are released to the cytosol and even to the extracellular space via exosomes, where they activate TLR3 in neighboring cells [13]. Moreover, under osteoarthritis-eliciting conditions, such as mitochondrial dysfunction, increased levels of reactive oxygen species (ROS), and DNA damage, mt-dsRNAs are released to the cytosol, where they activate PKR to promote chondrocyte death [10]. Lastly, activation of antiviral signaling by exogenous dsRNAs also results in aberrant accumulation and the subsequent cytosolic release of mt-dsRNAs to promote downstream immune signaling pathways [9]. In this context, mt-dsRNAs act as positive feedback factors to potentiate the antiviral signaling initiated by the exogenous dsRNAs [9].

The activation of antiviral signaling by mt-dsRNAs can be reduced by triggering autophagy [10]. A recent study reported that autophagy inducers such as torin-1 or metformin protected cells during mitochondrial stress by removing cytosolic mt-dsRNAs and subsequently preventing PKR activation. In addition, blocking the cytosolic release of mt-dsRNAs through the action of muscarinic agonist acetylcholine also protected cells from mt-dsRNA-mediated potentiation of antiviral signaling [9]. Based on this evidence, we asked whether enhancing mitochondrial function and protecting mitochondria from external stressors through antioxidant resveratrol (RES) could be another potential strategy for deactivating or preventing the activation of antiviral signaling. RES is a naturally occurring phenolic product abundantly found in grape skins and wines [14]. It is known to exhibit antioxidant properties both in vitro and in vivo settings through the activation of sirtulin 1 (SIRT1), mammalian nicotinamide adenine dinucleotide (NAD+)-dependent histone deacetylase that promotes mitochondrial function and protects the cells from oxidative stress [15]. Through SIRT1 activation, RES has demonstrated its ability as a neuroprotective agent by rescuing retinal neuronal cell death in diabetic mice [16]. Moreover, RES also shows antiviral effects in a wide range of human and animal viruses by interfering with the viral life cycle [17,18]. Considering the close association of mitochondrial function with diabetes and in response to viral infection, RES may also function to prevent the activation of antiviral signaling by mt-dsRNAs.

In this study, we investigated the potential of utilizing RES as a protective agent against immunogenic stressors that trigger the mt-dsRNA-mediated antiviral response. We first examined whether RES pretreatment affected the cellular response to exogenous dsRNAs by transfecting cells with polyinosinic-polycytidylic acid (poly I:C), a synthetic dsRNA that mimics viral infection. In particular, we examined the expression of key downstream effectors activated by poly I:C transfection using high-throughput sequencing. As an alternative method to induce the expression of mt-dsRNAs, we examined the effect of RES on mitochondrial stressor oligomycin A (OA). As a comparison, we used tunicamycin (TN) to examine the effect of RES during endoplasmic reticulum (ER) stress. Collectively, our study demonstrates the potential use of RES in protecting cells from mt-dsRNA-mediated activation of antiviral signaling during immunogenic stress.

## 2. Results

### 2.1. RES Prevents the Autoimmune Response by Exogenous dsRNAs

We began our investigation by analyzing the effect of RES on cellular response to poly I:C stimulation. We recently reported that transfecting poly I:C to human salivary gland acinar cells (NS-SV-AC) increased cellular mt-dsRNA levels and promoted the cytosolic release of mt-dsRNAs [9]. These mt-dsRNAs in the cytosol were then recognized by PKR and activated the kinase to potentiate the antiviral response initiated by poly I:C [9]. Of note, the poly I:C stimulation of salivary gland cells is commonly used to study the aberrant immune activation that drives the development of primary Sjögren’s syndrome (SS), a systemic autoimmune disease characterized by dysfunction in exocrine glands [9,19,20,21]. Consistent with our in vitro data, elevated mt-dsRNA expression was also observed in the saliva and tear samples of SS patients, and the expression of mt-dsRNA correlated with secretory dysfunction in patients [9]. Considering that antioxidant RES administration resulted in significant improvement in the salivary gland function of the non-obese diabetic mice (an in vivo model of SS) [22], we asked whether RES could counter the cellular response to poly I:C in NS-SV-AC cells at the molecular level.

**Figure 1:**
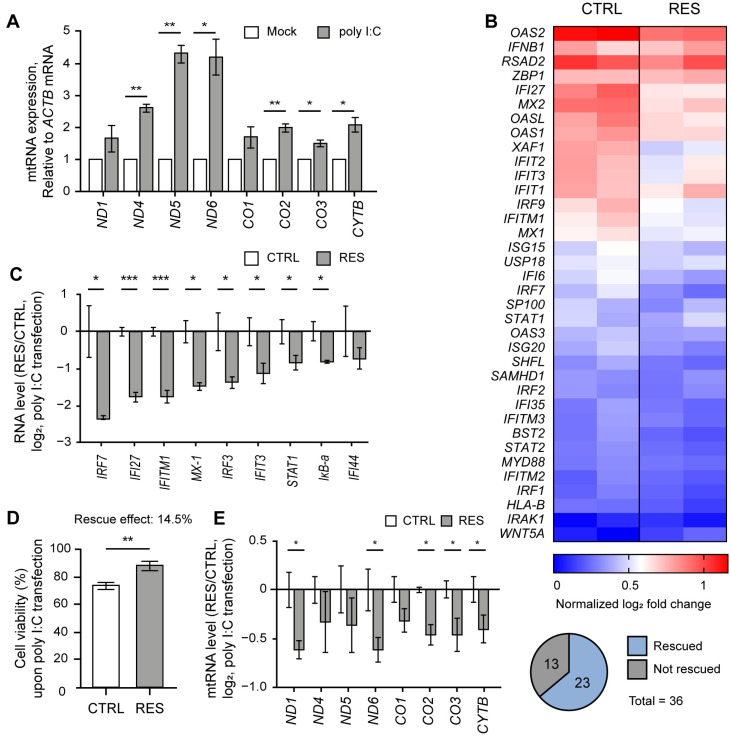
RES alleviated the IFN response by poly I:C. (**A**) The expression of mtRNAs in NS-SV-AC cells upon poly I:C transfection. (**B**) Heatmap of mRNA-seq results upon poly I:C transfection with or without RES pretreatment for type I IFN genes. The two columns represent log_2_ fold changes of two biological replicates. (**C**) The ratios of ISG induction upon poly I:C transfection between RES-pretreated and control samples. (**D**) The APH assay was used to measure the effect of RES pretreatment on cell viability. *n* = 4 and error bars are s.e.m. (**E**) The ratios of mtRNAs induction upon poly I:C transfection between RES-pretreated and control samples. For ratios, Cq values were first normalized to that of *ACTB* mRNA. For untreated samples, the values were normalized to RNAs from control cells without poly I:C transfection. Similarly, RNAs from RES-pretreated cells with poly I:C transfection were normalized separately from those from RES-treated cells without poly I:C. Unless mentioned otherwise, three independent experiments were carried out, and error bars denote s.e.m. All statistical significances were calculated using one-tailed Student’s *t*-tests; * *p* ≤ 0.05, ** *p* ≤ 0.01, and *** *p* ≤ 0.001.

Similar to our previous study [9], we cultured NS-SV-AC cells as 3D spheroids by employing a poly(2,4,6,7-tetravinyl-2,4,6,8-tetramethyl cyclotetrasiloxane) (pV4D4) functional polymer thin-film platform to consider the physiology of exocrine glands [23]. First, we confirmed that poly I:C stimulation led to the elevation of mtRNA transcripts in NS-SV-AC spheroids (Figure 1A). We then performed mRNA-seq analysis on NS-SV-AC spheroids transfected with poly I:C and showed significant induction of several IFN-stimulated genes (ISGs) relevant to SS, such as *MX2*, *MX1*, and *IFI27* [24,25] (Figure 1B, left columns). The degree of these ISG inductions was then compared with spheroids that were pretreated with RES 24 h prior to poly I:C transfection (Figure 1B, right columns). Here, we pretreated cells with 20 μM of RES because previous studies reported that low doses of RES ranging from 3–30 μM protected cells from oxidative stress by decreasing mitochondrial ROS production while increasing the expression of SIRT1 and its downstream antioxidant proteins, such as peroxisome proliferator-activated receptor gamma coactivator-1α (PGC-1α) and nuclear respiratory factor 1 (NRF-1) [26,27,28,29,30,31].

According to our mRNA-seq result, we found that out of 36 genes relevant to type I IFN, 23 genes showed more than a 1.5-fold degree of reduced induction, with *p*-values less than 0.05 (Figure 1B). Of note, we consider a gene “rescued” when the effects of poly I:C-triggered mtRNAs and ISG induction were reversed via RES treatment. We further confirmed the mRNA-seq results using RT-qPCR and validated that eight out of nine genes showed a statistically significant reduction in expression (Figure 1C). Next, we assembled the top 200 genes that showed a statistical rescue effect upon RES treatment, and performed gene ontology (GO) analysis to reveal that genes related to an antiviral immune response (both type I and type II IFN responses) were restored in RES-pretreated spheroids (Figure S1A,B). In addition to IFN responses, RES also reversed gene expressions related to chemoattractant, lymphocyte chemotaxis, and regulation of viral genome replication, indicating that RES pretreatment countered the poly I:C-triggered gene expression changes globally (Figure S1B).

As the increased dsRNA expression by poly I:C transfection also initiated apoptotic programs, we performed an acid phosphatase assay (APH) to examine the effect of RES on cell proliferation (Figure 1D). While poly I:C transfection decreased cell viability by 15%, this reduction was restored by 14.5% in cells pretreated with RES. Ultimately, we investigated whether the apparent rescue effect of RES pretreatment was attributable to changes in mtRNA expression. Similar to ISG induction, five out of eight mtRNAs showed a statistically significant rescue effect upon RES pretreatment prior to poly I:C transfection (Figure 1E).

**Figure 2:**
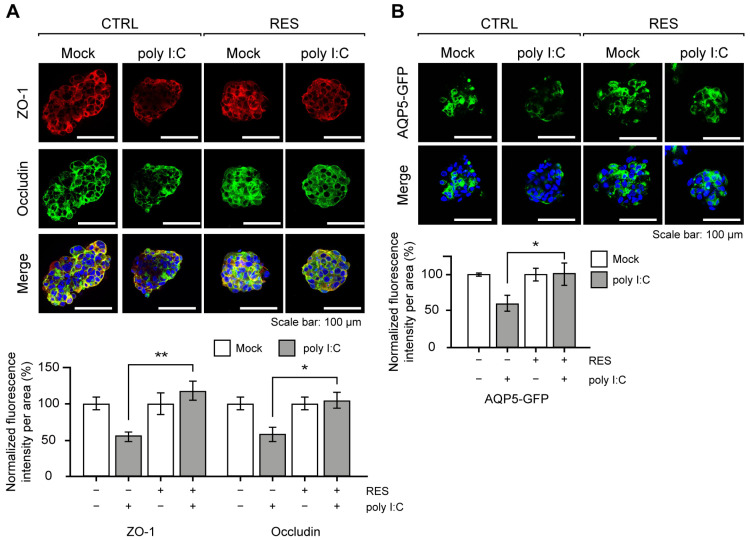
RES pretreatment rescued SS-related protein expressions. (**A**) Representative images of ZO-1 and occludin upon poly I:C transfection with or without RES pretreatment. The quantification of ZO-1 and occludin expressions is presented as the mean fluorescence intensity per spheroid. Intensities were normalized to the data from each experimental group cultured without poly I:C. *n* = 3 and error bars are s.e.m. (**B**) Visualization of GFP-tagged AQP5 expression upon poly I:C transfection. The scale bar represents 100 μm. The mean fluorescence intensities were normalized to the data from each experimental group cultured without poly I:C. In the bar graphs, the “–“ sign indicates RES-untreated/Mock-transfected, whereas the “+” sign indicates RES-treated/poly I:C-transfected. *n* = 3 and error bars are s.e.m. All statistical significances were calculated using one-tailed Student’s *t*-tests; * *p* ≤ 0.05, ** *p* ≤ 0.01.

The poly I:C stimulation in NS-SV-AC spheroids is known to mimic the glandular characteristics of SS, such as the disruption of tight junction complex (TJC) proteins and water channel proteins [32,33]. Based on the previous reports [32,33,34], we examined the expression patterns of two TJC proteins, Zonula occludens-1 (ZO-1) and occludin, as well as aquaporin-5 (AQP5) water channel protein as readouts for the effects of RES. Of note, we generated and analyzed NS-SV-AC cells with a stable expression of AQP5-GFP under the constitutive promoter for robust analysis of AQP5 expression in the 3D spheroids. Upon RES pretreatment, we found that the expression levels of the aforementioned proteins were all restored nearly completely (Figure 2A,B). Quantification of the image data clearly showed that the signal intensities of both ZO-1 and occludin were not affected by poly I:C in the RES-pretreated cells. A similar effect was observed for AQP5.

### 2.2. Mitochondrial Stressor OA Induces mtRNA Expression

In addition to the role of RES in protecting cells from autoimmune responses to poly I:C [22,35,36,37], we asked whether the antioxidant could also rescue the downstream effects of other types of cellular stressors. For the rest of the investigation, we switched the experimental system to the HCT116 colorectal carcinoma cell line because ROS production and the subsequent oxidative stress are often associated with colorectal cancer progression [38]. In particular, metastasis of colorectal cancer is developed from various signaling pathways regulated by ROS production and oxidative stress [39,40,41]. Moreover, antioxidant and anti-inflammatory effects are becoming increasingly important in preventing colorectal cancer because oxidative stress can trigger fibrosis and tissue injuries in colorectal cancer patients with chronic inflammation in the gastrointestinal tract [40,42,43,44,45]. Therefore, we examined the effect of immunogenic stressors that could induce an inflammatory response in HCT116 cells and investigated whether RES could counter their downstream effects.

**Figure 3:**
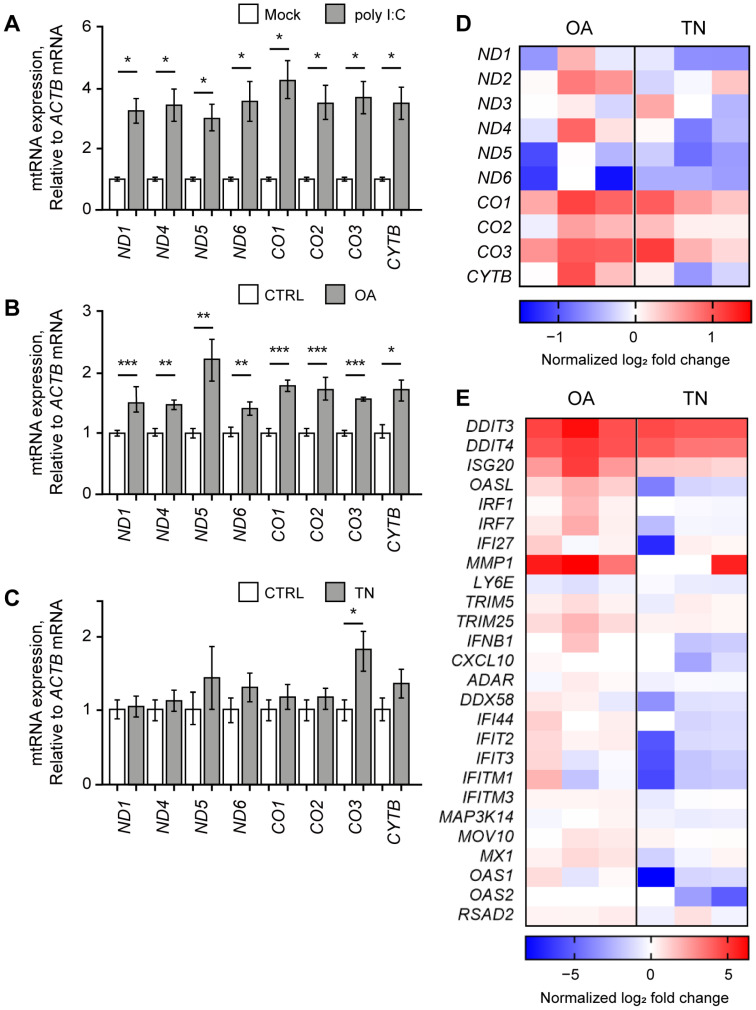
OA elevated mtRNA and ISG expressions. The expression of mtRNAs in HCT116 cells upon poly I:C transfection (**A**), OA treatment (**B**), or TN treatment (**C**). All Cq values are relative to that of *ACTB* mRNA. The values of poly I:C-transfected samples were normalized to the mock-transfected samples, and values of OA- or TN-treated samples were normalized to the DMSO-treated samples. (**D**,**E**) Heatmap of differentially expressed genes represents mtRNAs (**D**) and ISGs (**E**) upon OA or TN treatment. Each column represents the log_2_ fold change of three biological replicates with OA or TN treatment normalized by the control average. Unless mentioned otherwise, three independent experiments were carried out, and error bars denote s.e.m. All statistical significances were calculated using one-tailed Student’s *t*-tests; * *p* ≤ 0.05, ** *p* ≤ 0.01, and *** *p* ≤ 0.001.

Similar to our study using NS-SV-AC cells, we first analyzed the effect of poly I:C stimulation and found that the poly I:C resulted in significant upregulation of mtRNAs in HCT116 cells (Figure 3A). All examined mtRNAs showed at least a three-fold increase in expression seven h after poly I:C transfection. We then expanded our investigation to examine the potential rescue effect of RES on other immunogenic stressors that might exert their effects via mtRNAs. We chose OA and TN as two representative stressors that target mitochondria or ER, respectively. OA is an inhibitor of ATP synthase in the electron transfer system often used to induce mitochondrial dysfunction [10,46,47], and TN is known to induce ER stress by promoting the accumulation of unfolded proteins in the ER lumen [48,49,50]. With these stressors, we analyzed first whether they could induce the expression of mtRNAs, and then whether RES could counter the downstream effects of these stressors.

First, we treated HCT116 cells with OA and found that the expression levels of all examined mtRNAs were increased significantly (Figure 3B). This result is consistent with our recent report that mitochondrial dysfunction under osteoarthritis-eliciting conditions resulted in the elevation of mt-dsRNA levels and subsequent PKR activation [10]. Next, we treated HCT116 cells with TN, but we could not observe any significant change in mtRNA expression except for *CO3* mtRNA (Figure 3C).

We further analyzed the cellular response to OA or TN treatment through mRNA-seq and found that the increase in mtRNA expression is more apparent in OA-treated cells than in TN-treated cells (Figure 3D). Except for *ND5* and *ND6*, mtRNAs showed an increase in expression upon OA treatment (Figure 3D, left columns). Moreover, OA treatment induced the expression of 24 out of 26 ISGs, including *DDIT4*, *ISG20*, *OASL*, *IRF1*, *IRF7*, *IFI27*, and *MMP1*, as shown in the left three columns of Figure 3E. Clearly, increased expression of mtRNAs correlated with the IFN response assessed by the induction of ISGs. The GO analysis of OA-treated cells revealed the activation of pathways related to programmed cell death, including the intrinsic apoptotic signaling pathway, apoptotic signaling pathway, and regulation of the apoptotic signaling pathway (Figure S2A). Other pathways that were significantly upregulated upon OA treatment included a response to the ER stress, response to extracellular stimulus, epithelial cell migration, and a response to decreased oxygen levels (Figure S2B).

In the case of TN-treated cells, the mRNA-seq analysis revealed decreased levels of many mtRNAs, such as *ND1*, *ND4*, *ND5*, *ND6*, and *CYTB* (Figure 3D, right columns). Notably, the overall change in mtRNA expression was negligible in TN-treated cells compared to OA-treated cells. Moreover, 20 out of 26 ISG levels were either unaffected or slightly downregulated upon TN treatment (Figure 3E, right columns), thereby suggesting that TN has only minor effects on mtRNA expression and the following ISG induction. While changes in ISG levels upon TN treatment were not so evident, our mRNA-seq data clearly revealed a significant increase in the expression of genes related to ER stress, such as *ATF4*, *XBP1*, and *ERN1* (Figure S3A). Indeed, our GO analysis revealed that pathways related to cellular response to chemical stress, response to ER stress, and the regulation of the apoptotic process were significantly activated (Figure S3B). More specifically, pathways related to unfolded proteins, including the regulation of peptidyl-tyrosine phosphorylation, the protein-containing complex assembly, and peptidase activity, were upregulated upon TN treatment (Figure S3C). Overall, our data suggest that although TN treatment did stimulate unfolded protein accumulation to induce ER stress and subsequent apoptosis, such a response was not accompanied by an increase in mtRNA or ISG expression.

### 2.3. RES Protects Cells from OA-Induced IFN Response

**Figure 4:**
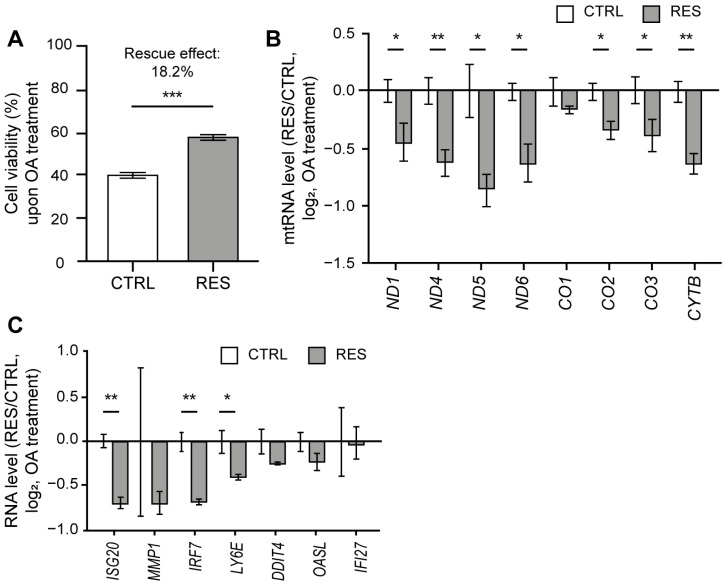
RES treatment alleviated cell death and IFN response to OA. (**A**) Cell viability upon OA treatment with or without RES treatment measured using a SRB assay. (**B**) The ratios of mtRNA induction upon OA treatment between RES-treated and control samples. (**C**) The ratios of ISG induction upon OA treatment between RES-treated and control samples. For ratios, Cq values were first normalized to that of *ACTB* mRNA. For untreated samples, the values were normalized to RNAs from control cells without OA treatment. Similarly, RNAs from cells co-treated with RES and OA were normalized separately from those from cells treated only with RES without OA. Unless mentioned otherwise, three independent experiments were carried out, and error bars denote s.e.m. All statistical significances were calculated using one-tailed Student’s *t*-tests; * *p* ≤ 0.05, ** *p* ≤ 0.01, and *** *p* ≤ 0.001.

As OA treatment resulted in the induction of mtRNA and ISG expressions, we asked whether RES treatment could counter such downstream responses, thereby protecting cells from mitochondrial stress by OA. We first analyzed the effect of RES treatment on OA-induced cell death through a sulforhodamine B (SRB) assay. The OA treatment decreased the cell viability by 61% (Figure 4A), which is most likely attributable to the apoptosis induced by ATP synthase inhibition [51]. Interestingly, the reduction in cell viability was restored by 18.2% upon RES treatment (Figure 4A). Of note, for the case of OA, we observed a more promising rescue effect when RES was co-treated with OA rather than pretreated 24 h prior to the OA treatment. Therefore, for the subsequent analysis, we examined the rescue effect of RES co-treated with OA.

The expression of mtRNA was significantly decreased when RES was co-treated with OA, with the exception of just one mtRNA (*CO1*) (Figure 4B). Following the decrease in mtRNA expression, we examined selected ISGs that showed increased expression upon OA treatment. All tested ISGs showed reduced expression when RES was treated with OA (Figure 4C). Of note, we could not observe a statistically significant change in many ISGs because the degree of induction in control cells was quite variable. However, we consistently observed a reduced degree of induction for ISGs when cells were co-treated with RES compared to those treated with OA alone. Hence, our data demonstrate that RES is able to lessen the ISG response to mitochondrial stress by OA.

Unlike OA, RES did not induce a significant rescue effect on TN-treated cells (Figure S4). Cell viability was decreased by 44% upon TN treatment, but no statistically significant rescue effect was observed when RES was co-treated with TN (Figure S4A). Moreover, the mtRNA expression did not show statistically significant change upon co-treatment of RES and TN, with the exception of *CO3* (Figure S4B). Similarly, RES treatment with TN did not lead to significant changes in ISG expression, with the only exception being *ISG20* (Figure S4C). Lastly, we examined whether RES could rescue the ER stress response via TN. When we analyzed the changes in expression levels of key ER stress response genes, such as *IRE1*, *HSPA5*, *GADD34*, *XBP1*, and *CHOP*, we found that RES treatment did not affect their induction via TN treatment in a consistent manner (Figure S4D). The expression levels of *PERK* and *CNAX* mRNA did decrease with RES treatment, but the overall effect was marginal. Altogether, our data suggest that antioxidant RES does not protect cells from ER stress via TN treatment.

## 3. Discussion

Our study provides a potential application of RES in ameliorating aberrant immune activation that involves mtRNAs. We first showed that RES pretreatment could protect the glandular phenotypes of autoimmune disease SS triggered by the introduction of poly I:C and subsequent activation of antiviral signaling. Notably, this protective effect of RES was not limited to type I IFN response by poly I:C but also extended to other molecular phenotypes, such as the disruption in TJC and water channel protein expression patterns. In this context, our results are in line with the effect of RES on improving salivary dysfunction in the mouse model of SS [22]. Further investigation of how RES is involved in mtRNA regulation may help to provide an improved treatment strategy for autoimmune SS patients.

To investigate the effect of RES in other pathophysiological conditions, we employed two additional cellular stressors in a colorectal cancer cell line. Considering the active role of ROS and the consequential oxidative stress in the progression and metastasis of colorectal cancers [39,40,41], we examined whether RES could exert antioxidant and anti-inflammatory effects to alleviate immunogenic stresses palpable in colorectal cancer, such as mitochondrial and ER stresses. In our study, we found that RES protected cells from mitochondrial dysfunction via OA treatment, which led to the elevation of both mtRNA and ISG levels. On the contrary, RES did not protect cells from TN-triggered ER stress, which only showed a marginal induction of ISGs and mtRNAs. Although our analyses were limited to three different immunogenic stressors, our data suggest that RES might attenuate the cellular response to stressors involving mtRNAs.

To the best of our knowledge, there have not been many attempts to understand the role of RES on mtRNA expression. Previous studies reported that a low dosage of RES in the range of 3–30 μM resulted in an increased number of mitochondria, mtDNA copies, mitochondrial transcription factor (TFAM), and proteins encoded by the mitochondrial genome to support mitochondrial function [28,52,53]. Based on this evidence, we presume that in healthy conditions, the antioxidant RES may play a supporting role to ensure active mitochondrial biogenesis. On the contrary, under immunogenic stress conditions that accompany increased mtRNA expression, RES may function to protect cells by downregulating mtRNAs.

The potential mechanism behind the role of RES in reducing the mtRNA-mediated immune response can be explained by the protective effects of RES on mitochondria [54,55,56,57]. RES exhibits both antioxidant and anti-inflammatory properties [58,59,60] by modulating signaling pathways involved in improving mitochondrial structure and function [61,62]. RES can also activate AMP-activated protein kinase (AMPK) and PGC-1α via SIRT1 to initiate mitochondrial biogenesis and protect cells from a decreased metabolic rate [63,64,65]. Moreover, PGC-1α activates mitochondrial transcription factors such as NRF-1 and estrogen-related receptor α (ERRα) to promote the expression of nucleus-encoded mitochondrial proteins [66,67,68], including TFAM, TFB1M, and TFB2M, that participate in the regulation of mtDNA homeostasis and mtRNA synthesis [69,70,71]. Lastly, RES can activate sirtuin 3 (SIRT3) to stimulate the tricarboxylic acid (TCA) cycle, oxidative phosphorylation, and fatty acid oxidation, thus enhancing mitochondrial function [72]. Altogether, RES protects mitochondria under stress by reinforcing mitochondrial function and preventing aberrant mtRNA induction.

An alternative explanation is the ability of RES to modulate the antiviral signaling pathways. A myriad of previous studies reported that RES has antiviral properties by reducing unnecessary ROS production [73] and by inhibiting the nuclear factor kappa-light-chain-enhancer of activated B cells (NF-κB) and extracellular signal-regulated kinase/mitogen-activated protein kinase (ERK/MAPK) pathways [74,75]. Considering that NF-κB promotes ISG induction through the activation of the interferon response and Janus kinase 1/signal transducer and is the activator of the transcription (JAK1/STAT) pathway [76], the observed protective effect of RES can be explained by its ability to prevent the activation of the JAK1/STAT pathway. Consistent with this, we previously showed that poly I:C-driven elevation of mt-dsRNA expression was abolished when the small chemical inhibitor for JAK1 upadacitinib was treated [9]. Overall, the antioxidant may contribute to lessening the mtRNA-mediated cellular stress response by inhibiting the JAK1/STAT pathway activated by the immunogenic stressors.

**Figure 5:**
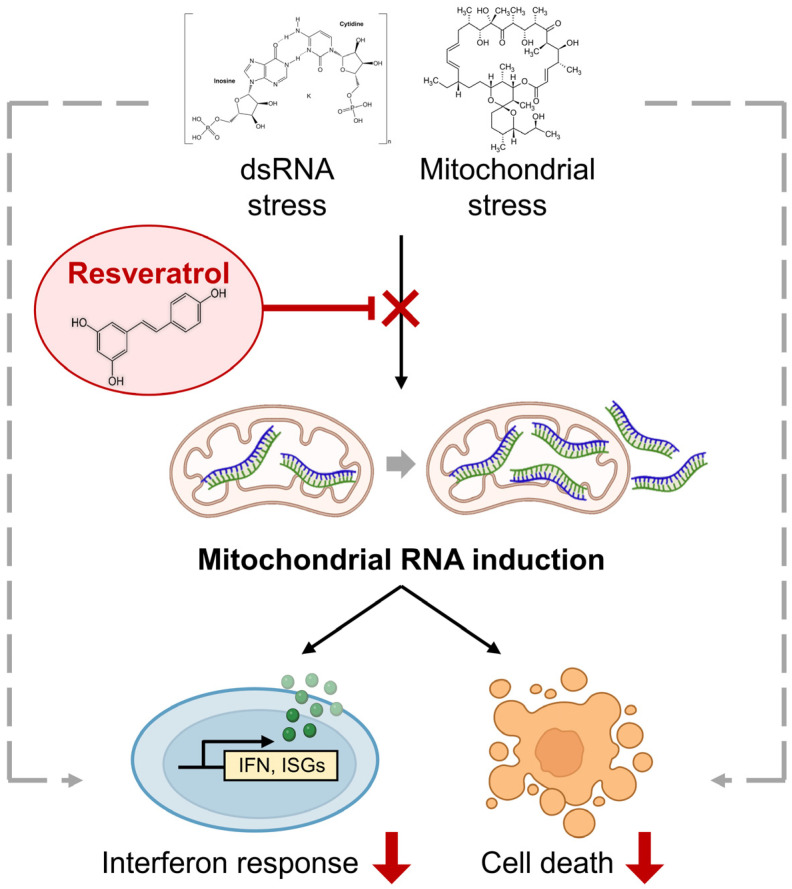
The rescue effect of RES on cellular response to immunogenic stresses. Two immunogenic stressors, poly I:C stimulation and OA, exert their downstream effects partly via mtRNAs. By promoting mitochondrial function and preventing mtRNA induction, an antioxidant RES can counter the molecular and cellular responses to these stressors.

In sum, our study highlights the importance of antioxidant RES in attenuating mtRNA-mediated cellular stress response to at least two immunogenic stressors (Figure 5). This knowledge will have potential implications for the design of targeted therapies to remove accumulated mtRNAs or facilitate the mtRNA decay to ultimately alleviate stress response arising from various pathological conditions.

## 4. Materials and Methods

### 4.1. Cell Culture and Chemical Treatment

A SV40-immortalized NS-SV-AC human salivary gland acinar cell line was kindly provided by Professor Masayuki Azuma (Department of Oral Medicine, the University of Tokushima Graduate Faculty of Dentistry, Tokushima, Japan). NS-SV-AC cells were grown in Keratinocyte-SFM (Serum-free media; GIBCO, Waltham, MA, USA) supplemented with 10% (*v*/*v*) heat-inactivated fetal bovine serum (FBS; Welgene, Gyeongsan, South Korea) and 1% (*v*/*v*) 100× penicillin–streptomycin (GIBCO, Waltham, MA, USA). pGFP-hAQP5-C1 plasmid, a kind gift from Professor Kyung Pyo Park (Seoul National University, Seoul, Republic of Korea), was used to develop the hAQP5-expressing NS-SV-AC cell line under the constitutive promoter (G418 100 μg/mL selection). HCT116 human colorectal cancer cells were grown in RPMI1640 (GIBCO) supplemented with 10% (*v*/*v*) FBS (Welgene). All cells were maintained at 37 °C in a humidified 5% CO_2_ atmosphere.

To induce dsRNA stress for NS-SV-AC cells, 20 μg/mL of poly I:C (Sigma Aldrich, St.Louis, MO, USA) was transfected using Lipofectamine 3000 (Thermo Fisher Scientific, Waltham, MA, USA) for 14 h following the manufacturer’s guide. For HCT116 cells obtained from the American Type Culture Collection (ATCC, Manassas, VA, USA), 10 μg/mL of poly I:C (Sigma Aldrich) was transfected using Lipofectamine 3000 (Thermo Fisher Scientific) for 7 h. As for the controlled mock transfection, Diethyl pyrocarbonate (DEPC)-treated water was used. In HCT116 cells, 10 μg/mL of OA or 5 μg/mL of TN was treated. For the control sample, the same volume of DMSO was treated.

RES stock solution of 20 mM was prepared by dissolving 4.565 g of RES powder (Sigma Aldrich, R5010) in 1 mL of DMSO. The stock solution was then aliquoted into small volumes (e.g., 20 μL) and stored at −20 °C until use. For NS-SV-AC cells, 20 μM of RES was pretreated for 24 h prior to poly I:C transfection. Upon poly I:C transfection, the same concentration of RES was treated once again. In HCT116 cells, 10 μM of RES was co-treated with 10 μg/mL of OA or 5 μg/mL of TN for 24 h. The same volume of DMSO was treated as the control in both cell lines.

### 4.2. Immunocytochemistry

Spheroids from pV4D4-coated plates were transferred to a 1.5 mL tube, and analysis for 2D culture was performed on a confocal dish (SPL). Cells were fixed in 4% (*w*/*v*) paraformaldehyde for 15 min at room temperature (RT). Fixed cells were then permeabilized with 0.3% (*w*/*v*) triton-X-100 (Sigma Aldrich) in Dulbecco’s phosphate-buffered saline (DPBS, Takara, Kusatsu, Japan) for 10 min at RT and blocked in 3% bovine serum albumin (BSA100; Bovogen, Keilor East, Australia) for 1 h. Cells were incubated with primary antibodies diluted in 1% BSA at a 1:100 ratio overnight at 4 °C. The primary antibodies used in this study include: occludin (Invitrogen, Carlsbad, CA, USA; OC-3F10) and ZO-1 (Cell Signaling Technology, Danvers, MA, USA; 8193S). Cells were then washed with DPBS and incubated with an Alexa fluor 488-conjugated anti-mouse secondary antibody (Thermo Fisher Scientific; A31570) and an Alexa fluor 555 conjugated anti-mouse secondary antibody (Thermo Fisher Scientific; A-21202) diluted at a 1:1000 ratio for 45 min at RT. DAPI was incubated for 5 min. All the washing steps for spheroids consist of centrifugation (5 min, 200 g) and supernatant discarding. Fluorescent images were obtained using a confocal laser-scanning microscope (LMS 880, Carl Zeiss, Oberkochen, Germany). The fluorescent intensity per area was quantified as the integrated density divided by the area of each spheroid and then normalized to the control without poly I:C transfection.

To visualize the expression levels of AQP5, NS-SV-AC cells with stable expression of GFP-AQP5 were grown on pV4D4-coated plates as 3D spheroids. The spheroids were then transferred to a 1.5 mL tube and fixed in 4% (*w*/*v*) paraformaldehyde for 15 min at RT. Fixed cells were permeabilized with 0.3% (*w*/*v*) triton-x-100 (Sigma Aldrich) in DPBS for 10 min at RT. Cells were then stained with DAPI for 5 min. Fluorescent images were obtained using a confocal laser-scanning microscope (LMS 880, Carl Zeiss, Oberkochen, Germany). The fluorescent intensity per area was quantified as described above.

### 4.3. RNA Extraction and Quantitative Real-Time PCR

To extract total RNAs, TRIzol (Ambion, Foster City, CA, USA) was added directly to the cell pellet obtained after centrifugation at 10,000× *g* for 30 s. After precipitation of the extracted nucleic acids, DNase I (Takara, Kusatsu, Japan) was treated to remove DNA, and purified RNA was reverse transcribed using RevertAid reverse transcriptase (Thermo Fisher Scientific, Waltham, MA, USA). For RNAs extracted from 3D spheroids, SuperScript IV Reverse Transcriptase (Invitrogen, Carlsbad, CA, USA) was used to synthesize cDNA. cDNA was amplified using SYBR Green PCR master mix (Thermo Fisher Scientific) and analyzed using the StepOnePlus real-time PCR system. Primer sequences used in this study are provided in Table S1.

### 4.4. Acid Phosphatase Assay

Cells (or spheroids) were centrifuged for 10 min at 400 g for spin down. After washing the pellets twice with DPBS, the supernatant was discarded to obtain the final volume of 100 μL. Then, 100 μL of APH assay buffer (0.1 M sodium acetate, 0.1% (*v*/*v*) triton-x-100, 2 mg/mL Immunopure PNPP (Sigma Aldrich) in deionized/distilled water) was added to each well and incubated for 90 min at 37 °C with 5% CO_2_. After incubation, 10 μL of 1 N NaOH was added, and absorption at 405 nm was analyzed on a microplate reader (BioTek, Winooski, VT, USA). For each experiment, cell viability was normalized to the data from the control group without poly I:C transfection.

### 4.5. Sulforhodamine B (SRB) Assay

To analyze cell viability after treatment of OA or TN with RES, cells were fixed with a 10% (*w*/*v*) trichloroacetic acid solution in distilled water for 1 h at 4 °C. Cells were washed with cold PBS 4 times and air-dried. Dried cells were stained with a 0.4% (*w*/*v*) SRB (Sulforhodamine B sodium salt, Sigma Aldrich, St.Louis, MO, USA) solution in 1% acetic acid for 30 min at RT. The cells were washed with 1% acetic acid 4 times while protected from light. The air-dried dye was eluted with a 10 mM Tris (Bio-basic, Markham, ON, Canada) solution (pH 10.5), and absorbance at 510 nm was measured using a Varioskan LUX multimode microplate reader (Thermo Fisher Scientific). Cell viability was normalized to the data from the control group without OA or TN treatment.

### 4.6. mRNA-seq Data Analysis

Read files were quality checked with FastQC (ver 0.11.5) (Babraham Institute, Cambridge, UK). For poly I:C transfection and RES treatment, reads were aligned to the human genome (hg38) using Hisat2 (ver 2.1.0, Johns Hopkins University, Baltimore, MD, USA) [77]. The aligned reads were quantified with StringTie (ver 2.0.4) (Johns Hopkins University, Baltimore, MD, USA) [78] using the human gene annotation file version 32 from Gencode as the index. For OA and TN treatment, reads were aligned to the human genome (hg38) using the STAR aligner (ver 2.7.9) (Cold Spring Harbor Laboratory, Cold Spring Harbor, NY, USA) [79]. Reads were simultaneously quantified using the STAR aligner’s gene counts option with human gene annotation file version 32 from Gencode as the index. Differentially expressed genes (DEGs) were searched with DESeq2 (ver 1.30.1) (European Molecular Biology Laboratory, Heidelberg, Germany) [80].

### 4.7. DEG Analysis

For DEG analysis, DESeq2 analysis [80] was performed separately in each of the DMSO- and RES-pretreated groups. Within each group, raw counts of two replicates with poly I:C transfection were compared against the raw counts of two replicates with the control group (i.e., DEPC pretreatment). For OA and TN treatment, three biological replicates were compared against three control replicates (i.e., DMSO treatment). The counts were normalized by DESeq2 during analysis. The analysis yielded three separate lists of DEGs, and the genes were ordered by the lowest adjusted *p*-values for further analysis.

### 4.8. GO Analysis of DEGs

Among genes that show log_2_ fold change with *p*-values less than 0.05, the values of DMSO (i.e., control) were subtracted from those of RES-pretreated RNAs to evaluate the degree of rescue in the induction of gene expression. The top 200 genes that show the rescue effects were assembled into a gene list for GO analysis. For OA and TN treatment, upregulated genes with log_2_ fold change over 0.585 compared to DMSO-treated samples, with *p*-values less than 0.05, were subtracted and analyzed. GO analysis was performed using ClueGo software (Cytoscape, v. 3.7.1, Institute for Systems biology, Seattle, WA, USA) [81].

### 4.9. Statistical Analysis

Quantitative RT-PCR data and APH assay results were analyzed using the one-tailed Student’s *t*-test. All data were biologically replicated at least three times. The error bars indicate the standard error of the mean. *p*-values ≤ 0.05 were regarded as statistically significant. * denotes *p*-values ≤ 0.05, ** is *p*-values ≤ 0.01, and *** is *p*-values ≤ 0.001.

## Data Availability

All data needed to evaluate the conclusions in the paper are present in the paper and/or the Supplemental Information. Further information and requests for resources and reagents should be directed to and will be fulfilled by the lead contact, Yoosik Kim (ysyoosik@kaist.ac.kr). Sequencing data that support the findings of this study are available in the GEO database under the accession numbers Gene Expression Omnibus: GSE168148 and GSE226549. No materials were generated in this study. This manuscript does not report the original code. The computational pipeline used in this study is open-sourced and available at: https://daehwankimlab.github.io/hisat2/, https://ccb.jhu.edu/software/stringtie/, and http://www.bioconductor.org/packages/release/bioc/html/DESeq2.html. Accessed on 12 November 2020.

## Supplementary Materials

The following supporting information can be downloaded at: https://www.mdpi.com/article/10.3390/ijms24087403/s1.

## References

[1] Mercer T.R., Neph S., Dinger M.E., Crawford J., Smith M.A., Shearwood A.M., Haugen E., Bracken C.P., Rackham O., Stamatoyannopoulos J.A. (2011). The human mitochondrial transcriptome. Cell.

[2] Trounce I. (2000). Genetic control of oxidative phosphorylation and experimental models of defects. Hum. Reprod..

[3] West A.P., Shadel G.S., Ghosh S. (2011). Mitochondria in innate immune responses. Nat. Rev. Immunol..

[4] Reikine S., Nguyen J.B., Modis Y. (2014). Pattern Recognition and Signaling Mechanisms of RIG-I and MDA5. Front. Immunol..

[5] Luna-Sanchez M., Bianchi P., Quintana A. (2021). Mitochondria-Induced Immune Response as a Trigger for Neurodegeneration: A Pathogen from Within. Int. J. Mol. Sci..

[6] Zhang W., Li G., Luo R., Lei J., Song Y., Wang B., Ma L., Liao Z., Ke W., Liu H. (2022). Cytosolic escape of mitochondrial DNA triggers cGAS-STING-NLRP3 axis-dependent nucleus pulposus cell pyroptosis. Exp. Mol. Med..

[7] Maekawa H., Inoue T., Ouchi H., Jao T.M., Inoue R., Nishi H., Fujii R., Ishidate F., Tanaka T., Tanaka Y. (2019). Mitochondrial Damage Causes Inflammation via cGAS-STING Signaling in Acute Kidney Injury. Cell Rep..

[8] Dhir A., Dhir S., Borowski L.S., Jimenez L., Teitell M., Rotig A., Crow Y.J., Rice G.I., Duffy D., Tamby C. (2018). Mitochondrial double-stranded RNA triggers antiviral signalling in humans. Nature.

[9] Yoon J., Lee M., Ali A.A., Oh Y.R., Choi Y.S., Kim S., Lee N., Jang S.G., Park S., Chung J.H. (2022). Mitochondrial double-stranded RNAs as a pivotal mediator in the pathogenesis of Sjögren’s syndrome. Mol. Ther. Nucleic Acids.

[10] Kim S., Lee K., Choi Y.S., Ku J., Kim H., Kharbash R., Yoon J., Lee Y.S., Kim J.H., Lee Y.J. (2022). Mitochondrial double-stranded RNAs govern the stress response in chondrocytes to promote osteoarthritis development. Cell Rep..

[11] Barshad G., Marom S., Cohen T., Mishmar D. (2018). Mitochondrial DNA Transcription and Its Regulation: An Evolutionary Perspective. Trends Genet..

[12] Kim Y., Park J., Kim S., Kim M., Kang M.G., Kwak C., Kang M., Kim B., Rhee H.W., Kim V.N. (2018). PKR Senses Nuclear and Mitochondrial Signals by Interacting with Endogenous Double-Stranded RNAs. Mol. Cell.

[13] Lee J.H., Shim Y.R., Seo W., Kim M.H., Choi W.M., Kim H.H., Kim Y.E., Yang K., Ryu T., Jeong J.M. (2020). Mitochondrial Double-Stranded RNA in Exosome Promotes Interleukin-17 Production Through Toll-Like Receptor 3 in Alcohol-associated Liver Injury. Hepatology.

[14] Yadav E., Yadav P., Khan M.M.U., Singh H., Verma A. (2022). Resveratrol: A potential therapeutic natural polyphenol for neurodegenerative diseases associated with mitochondrial dysfunction. Front. Pharmacol..

[15] Tamaki N., Cristina Orihuela-Campos R., Inagaki Y., Fukui M., Nagata T., Ito H.O. (2014). Resveratrol improves oxidative stress and prevents the progression of periodontitis via the activation of the Sirt1/AMPK and the Nrf2/antioxidant defense pathways in a rat periodontitis model. Free Radic. Biol. Med..

[16] Kim Y.H., Kim Y.S., Kang S.S., Cho G.J., Choi W.S. (2010). Resveratrol inhibits neuronal apoptosis and elevated Ca2+/calmodulin-dependent protein kinase II activity in diabetic mouse retina. Diabetes.

[17] Abba Y., Hassim H., Hamzah H., Noordin M.M. (2015). Antiviral Activity of Resveratrol against Human and Animal Viruses. Adv. Virol..

[18] van Brummelen R., van Brummelen A.C. (2022). The potential role of resveratrol as supportive antiviral in treating conditions such as COVID-19-A formulator’s perspective. Biomed. Pharmacother..

[19] Deshmukh U.S., Nandula S.R., Thimmalapura P.R., Scindia Y.M., Bagavant H. (2009). Activation of innate immune responses through Toll-like receptor 3 causes a rapid loss of salivary gland function. J. Oral Pathol. Med..

[20] Zhou J., Jin J.O., Du J., Yu Q. (2015). Innate Immune Signaling Induces IL-7 Production, Early Inflammatory Responses, and Sjogren’s-Like Dacryoadenitis in C57BL/6 Mice. Investig. Opthalmol. Vis. Sci..

[21] Jin J.O., Shinohara Y., Yu Q. (2013). Innate immune signaling induces interleukin-7 production from salivary gland cells and accelerates the development of primary Sjogren’s syndrome in a mouse model. PLoS ONE.

[22] Inoue H., Kishimoto A., Ushikoshi-Nakayama R., Hasaka A., Takahashi A., Ryo K., Muramatsu T., Ide F., Mishima K., Saito I. (2016). Resveratrol improves salivary dysfunction in a non-obese diabetic (NOD) mouse model of Sjogren’s syndrome. J. Clin. Biochem. Nutr..

[23] Edmondson R., Broglie J.J., Adcock A.F., Yang L. (2014). Three-dimensional cell culture systems and their applications in drug discovery and cell-based biosensors. Assay Drug Dev. Technol..

[24] He Y., Chen R., Zhang M., Wang B., Liao Z., Shi G., Li Y. (2022). Abnormal Changes of Monocyte Subsets in Patients With Sjogren’s Syndrome. Front. Immunol..

[25] Sequi-Sabater J.M., Beretta L. (2022). Defining the Role of Monocytes in Sjogren’s Syndrome. Int. J. Mol. Sci..

[26] Gao R.Y., Mukhopadhyay P., Mohanraj R., Wang H., Horvath B., Yin S., Pacher P. (2011). Resveratrol attenuates azidothymidine-induced cardiotoxicity by decreasing mitochondrial reactive oxygen species generation in human cardiomyocytes. Mol. Med. Rep..

[27] Ungvari Z., Labinskyy N., Mukhopadhyay P., Pinto J.T., Bagi Z., Ballabh P., Zhang C., Pacher P., Csiszar A. (2009). Resveratrol attenuates mitochondrial oxidative stress in coronary arterial endothelial cells. Am. J. Physiol. Heart Circ. Physiol..

[28] Csiszar A., Bagi Z., Feher A., Recchia F.A., Sonntag W.E., Ungvari Z., Pearson K., de Cabo R. (2011). Resveratrol confers endothelial protection via activation of the antioxidant transcription factor Nrf2. FASEB J..

[29] Danz E.D.B., Skramsted J., Henry N., Bennett J.A., Keller R.S. (2009). Resveratrol prevents doxorubicin cardiotoxicity through mitochondrial stabilization and the Sirt1 pathway. Free Radic. Biol. Med..

[30] Xu Y., Nie L., Yin Y.G., Tang J.L., Zhou J.Y., Li D.D., Zhou S.W. (2012). Resveratrol protects against hyperglycemia-induced oxidative damage to mitochondria by activating SIRT1 in rat mesangial cells. Toxicol. Appl. Pharmacol..

[31] Zhou X., Chen M., Zeng X., Yang J., Deng H., Yi L., Mi M.T. (2014). Resveratrol regulates mitochondrial reactive oxygen species homeostasis through Sirt3 signaling pathway in human vascular endothelial cells. Cell Death Dis..

[32] Steinfeld S., Cogan E., King L.S., Agre P., Kiss R., Delporte C. (2001). Abnormal distribution of aquaporin-5 water channel protein in salivary glands from Sjogren’s syndrome patients. Lab. Investig..

[33] Ewert P., Aguilera S., Alliende C., Kwon Y.J., Albornoz A., Molina C., Urzua U., Quest A.F., Olea N., Perez P. (2010). Disruption of tight junction structure in salivary glands from Sjogren’s syndrome patients is linked to proinflammatory cytokine exposure. Arthritis Rheum..

[34] Chivasso C., D’Agostino C., Parisis D., Soyfoo M.S., Delporte C. (2023). Involvement of aquaporin 5 in Sjogren’s syndrome. Autoimmun. Rev..

[35] Nazari-Khanamiri F., Ghasemnejad-Berenji M. (2022). Resveratrol may ameliorate rheumatoid arthritis via the STAT3/HIF-1/VEGF molecular pathway. J. Food Biochem..

[36] Oliveira A.L.B., Monteiro V.V.S., Navegantes-Lima K.C., Reis J.F., Gomes R.S., Rodrigues D.V.S., Gaspar S.L.F., Monteiro M.C. (2017). Resveratrol Role in Autoimmune Disease—A Mini-Review. Nutrients.

[37] Sheng S., Wang X., Liu X., Hu X., Shao Y., Wang G., Mao D., Li C., Chen B., Chen X. (2022). The role of resveratrol on rheumatoid arthritis: From bench to bedside. Front. Pharmacol..

[38] Trachootham D., Alexandre J., Huang P. (2009). Targeting cancer cells by ROS-mediated mechanisms: A radical therapeutic approach?. Nat. Rev. Drug. Discov..

[39] Arulselvan P., Wen C.C., Lan C.W., Chen Y.H., Wei W.C., Yang N.S. (2012). Dietary administration of scallion extract effectively inhibits colorectal tumor growth: Cellular and molecular mechanisms in mice. PLoS ONE.

[40] Arulselvan P., Fard M.T., Tan W.S., Gothai S., Fakurazi S., Norhaizan M.E., Kumar S.S. (2016). Role of Antioxidants and Natural Products in Inflammation. Oxid. Med. Cell. Longev..

[41] van der Stok E.P., Spaander M.C.W., Grunhagen D.J., Verhoef C., Kuipers E.J. (2017). Surveillance after curative treatment for colorectal cancer. Nat. Rev. Clin. Oncol..

[42] Fatfat M., Abou Merhi R., Rahal O., Stoyanovsky D.A., Zaki A., Haidar H., Kagan V.E., Gali-Muhtasib H., Machaca K. (2014). Copper chelation selectively kills colon cancer cells through redox cycling and generation of reactive oxygen species. BMC Cancer.

[43] Redondo-Blanco S., Fernandez J., Gutierrez-del-Rio I., Villar C.J., Lombo F. (2017). New Insights toward Colorectal Cancer Chemotherapy Using Natural Bioactive Compounds. Front. Pharmacol..

[44] Xia C., Meng Q., Lin L.Z., Rojanasakul Y., Wang X.R., Jiang B.H. (2007). Reactive oxygen species regulate angiogenesis and tumor growth through vascular endothelial growth factor. Cancer Res..

[45] Stone W.L., Krishnan K., Campbell S.E., Palau V.E. (2014). The role of antioxidants and pro-oxidants in colon cancer. World J. Gastrointest. Oncol..

[46] Walker J.E., Lutter R., Dupuis A., Runswick M.J. (1991). Identification of the subunits of F1F0-ATPase from bovine heart mitochondria. Biochemistry.

[47] Vaamonde-Garcia C., Loureiro J., Valcarcel-Ares M.N., Riveiro-Naveira R.R., Ramil-Gomez O., Hermida-Carballo L., Centeno A., Meijide-Failde R., Blanco F.J., Lopez-Armada M.J. (2017). The mitochondrial inhibitor oligomycin induces an inflammatory response in the rat knee joint. BMC Musculoskelet. Disord..

[48] Wu H., Meng Z., Jiao Y., Ren Y., Yang X., Liu H., Wang R., Cui Y., Pan L., Cao Y. (2020). The endoplasmic reticulum stress induced by tunicamycin affects the viability and autophagy activity of chondrocytes. J. Clin. Lab. Anal..

[49] Shen M., Wang L., Guo X., Xue Q., Huo C., Li X., Fan L., Wang X. (2015). A novel endoplasmic reticulum stress-induced apoptosis model using tunicamycin in primary cultured neonatal rat cardiomyocytes. Mol. Med. Rep..

[50] Guha P., Kaptan E., Gade P., Kalvakolanu D.V., Ahmed H. (2017). Tunicamycin induced endoplasmic reticulum stress promotes apoptosis of prostate cancer cells by activating mTORC1. Oncotarget.

[51] Matsuyama S., Llopis J., Deveraux Q.L., Tsien R.Y., Reed J.C. (2000). Changes in intramitochondrial and cytosolic pH: Early events that modulate caspase activation during apoptosis. Nat. Cell Biol..

[52] Motlagh Scholle L., Schieffers H., Al-Robaiy S., Thaele A., Dehghani F., Lehmann Urban D., Zierz S. (2020). The Effect of Resveratrol on Mitochondrial Function in Myoblasts of Patients with the Common m.3243A>G Mutation. Biomolecules.

[53] Ferretta A., Gaballo A., Tanzarella P., Piccoli C., Capitanio N., Nico B., Annese T., Di Paola M., Dell’aquila C., De Mari M. (2014). Effect of resveratrol on mitochondrial function: Implications in parkin-associated familiar Parkinson’s disease. Biochim. Biophys. Acta.

[54] Ungvari Z., Sonntag W.E., de Cabo R., Baur J.A., Csiszar A. (2011). Mitochondrial protection by resveratrol. Exerc. Sport Sci. Rev..

[55] Jardim F.R., de Rossi F.T., Nascimento M.X., da Silva Barros R.G., Borges P.A., Prescilio I.C., de Oliveira M.R. (2018). Resveratrol and Brain Mitochondria: A Review. Mol. Neurobiol..

[56] de Oliveira M.R., Nabavi S.F., Manayi A., Daglia M., Hajheydari Z., Nabavi S.M. (2016). Resveratrol and the mitochondria: From triggering the intrinsic apoptotic pathway to inducing mitochondrial biogenesis, a mechanistic view. Biochim. Biophys. Acta.

[57] Zhou J., Yang Z., Shen R., Zhong W., Zheng H., Chen Z., Tang J., Zhu J. (2021). Resveratrol Improves Mitochondrial Biogenesis Function and Activates PGC-1alpha Pathway in a Preclinical Model of Early Brain Injury Following Subarachnoid Hemorrhage. Front. Mol. Biosci..

[58] Wolter F., Stein J. (2002). Biological activities of resveratrol and its analogs. Drug Future.

[59] Ghanim H., Sia C.L., Korzeniewski K., Lohano T., Abuaysheh S., Marumganti A., Chaudhuri A., Dandona P. (2011). A resveratrol and polyphenol preparation suppresses oxidative and inflammatory stress response to a high-fat, high-carbohydrate meal. J. Clin. Endocrinol. Metab..

[60] Brasnyo P., Molnar G.A., Mohas M., Marko L., Laczy B., Cseh J., Mikolas E., Szijarto I.A., Merei A., Halmai R. (2011). Resveratrol improves insulin sensitivity, reduces oxidative stress and activates the Akt pathway in type 2 diabetic patients. Br. J. Nutr..

[61] Das S., Das D.K. (2007). Anti-inflammatory responses of resveratrol. Inflamm. Allergy Drug Targets.

[62] Csaki C., Keshishzadeh N., Fischer K., Shakibaei M. (2008). Regulation of inflammation signalling by resveratrol in human chondrocytes in vitro. Biochem. Pharmacol..

[63] Higashida K., Kim S.H., Jung S.R., Asaka M., Holloszy J.O., Han D.H. (2013). Effects of resveratrol and SIRT1 on PGC-1alpha activity and mitochondrial biogenesis: A reevaluation. PLoS Biol..

[64] Csiszar A., Zhang H.R., Zhang C.H., Ungvari Z. (2010). Resveratrol induces mitochondrial biogenesis in endothelial cells. FASEB J..

[65] Price N.L., Gomes A.P., Ling A.J.Y., Duarte F.V., Martin-Montalvo A., North B.J., Agarwal B., Ye L., Ramadori G., Teodoro J.S. (2012). SIRT1 Is Required for AMPK Activation and the Beneficial Effects of Resveratrol on Mitochondrial Function. Cell Metab..

[66] Virbasius C.M.A., Virbasius J.V., Scarpulla R.C. (1993). Nrf-1, an Activator Involved in Nuclear-Mitochondrial Interactions, Utilizes a New DNA-Binding Domain Conserved in a Family of Developmental Regulators. Genes Dev..

[67] Anderson R., Prolla T. (2009). PGC-1alpha in aging and anti-aging interventions. Biochim. Biophys. Acta.

[68] Schreiber S.N., Knutti D., Brogli K., Uhlmann T., Kralli A. (2003). The transcriptional coactivator PGC-1 regulates the expression and activity of the orphan nuclear receptor estrogen-related receptor alpha (ERRalpha). J. Biol. Chem..

[69] Canugovi C., Maynard S., Bayne A.C., Sykora P., Tian J., de Souza-Pinto N.C., Croteau D.L., Bohr V.A. (2010). The mitochondrial transcription factor A functions in mitochondrial base excision repair. DNA Repair.

[70] Fisher R.P., Clayton D.A. (1988). Purification and characterization of human mitochondrial transcription factor 1. Mol. Cell. Biol..

[71] Picca A., Lezza A.M.S. (2015). Regulation of mitochondrial biogenesis through TFAM-mitochondrial DNA interactions Useful insights from aging and calorie restriction studies. Mitochondrion.

[72] Brenmoehl J., Hoeflich A. (2013). Dual control of mitochondrial biogenesis by sirtuin 1 and sirtuin 3. Mitochondrion.

[73] De Leo A., Arena G., Lacanna E., Oliviero G., Colavita F., Mattia E. (2012). Resveratrol inhibits Epstein Barr Virus lytic cycle in Burkitt’s lymphoma cells by affecting multiple molecular targets. Antivir. Res..

[74] Chen X.Q., Qiao H.S., Liu T.X., Yang Z.Y., Xu L.F., Xu Y.X., Ge H.M., Tan R.X., Li E.G. (2012). Inhibition of herpes simplex virus infection by oligomeric stilbenoids through ROS generation. Antivir. Res..

[75] Faith S.A., Sweet T.J., Bailey E., Booth T., Docherty J.J. (2006). Resveratrol suppresses nuclear factor-kappaB in herpes simplex virus infected cells. Antivir. Res..

[76] Peisley A., Hur S. (2013). Multi-level regulation of cellular recognition of viral dsRNA. Cell. Mol. Life Sci..

[77] Kim D., Paggi J.M., Park C., Bennett C., Salzberg S.L. (2019). Graph-based genome alignment and genotyping with HISAT2 and HISAT-genotype. Nat. Biotechnol..

[78] Pertea M., Pertea G.M., Antonescu C.M., Chang T.C., Mendell J.T., Salzberg S.L. (2015). StringTie enables improved reconstruction of a transcriptome from RNA-seq reads. Nat. Biotechnol..

[79] Dobin A., Davis C.A., Schlesinger F., Drenkow J., Zaleski C., Jha S., Batut P., Chaisson M., Gingeras T.R. (2013). STAR: Ultrafast universal RNA-seq aligner. Bioinformatics.

[80] Love M.I., Huber W., Anders S. (2014). Moderated estimation of fold change and dispersion for RNA-seq data with DESeq2. Genome Biol..

[81] Bindea G., Mlecnik B., Hackl H., Charoentong P., Tosolini M., Kirilovsky A., Fridman W.H., Pages F., Trajanoski Z., Galon J. (2009). ClueGO: A Cytoscape plug-in to decipher functionally grouped gene ontology and pathway annotation networks. Bioinformatics.

