# Supplementary material for: Resveratrol Attenuates the Mitochondrial RNA-Mediated Cellular Response to Immunogenic Stress
Source: Int J Mol Sci. 2023 Apr 17;24(8):7403. doi: 10.3390/ijms24087403 (PMC10138523; doi:10.3390/ijms24087403)
Supplement: Supplementary file 1 [file ijms-24-07403-s001.zip › ijms-2278852-supplementary.pdf]

SUPPLEMENTARY MATERIAL

Figure S1. The GO analysis of RES pretreated samples with poly I:C transfection.

Figure S2. The GO analysis of HCT116 cells treated with OA.

Figure S3. The TN treatment activates apoptotic and ER stress pathways.

Figure S4. The RES treatment does not rescue ER stress upon TN treatment.

Table S1. Primer sequences for RT-qPCR.

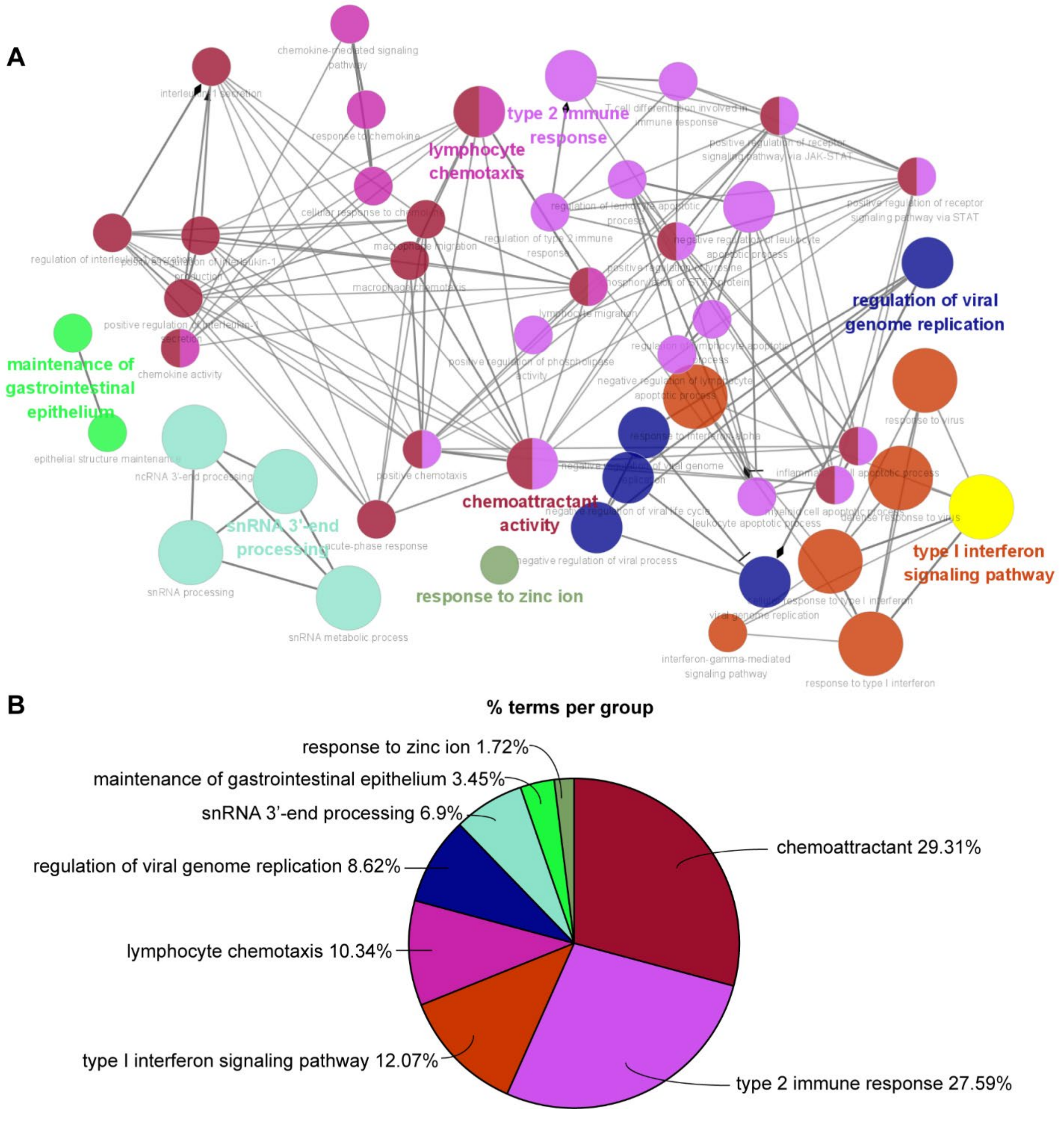

Figure S1. The GO analysis of RES pretreated samples with poly I:C transfection. (A) The GO of the top 200 genes whose $\log_2$ fold change upon poly I:C transfection was rescued by RES-pretreatment in NS-SV-AC cells. (B) A pie chart showing the class of genes rescued by RES-pretreatment upon poly I:C transfection in RES-pretreated NS-SV-AC cells.

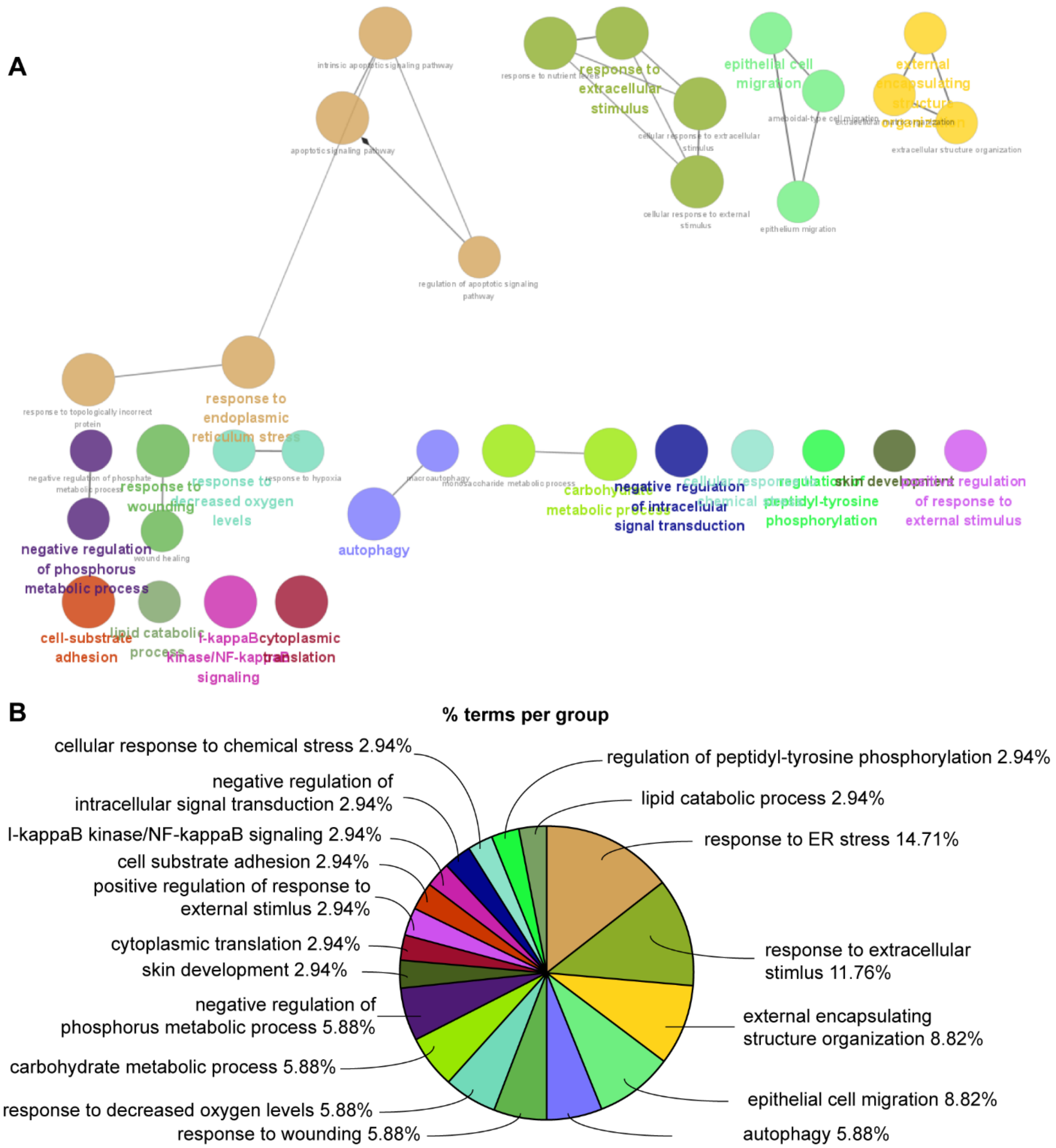

Figure S2. The GO analysis of HCT116 cells treated with OA. (A) The GO analysis of OA-treated samples. Genes whose $\log_2$ fold change was upregulated over 0.585 and p-value less than 0.05 upon OA treatment were analyzed. (B) A pie chart showing the class of genes whose expression was upregulated by OA treatment.

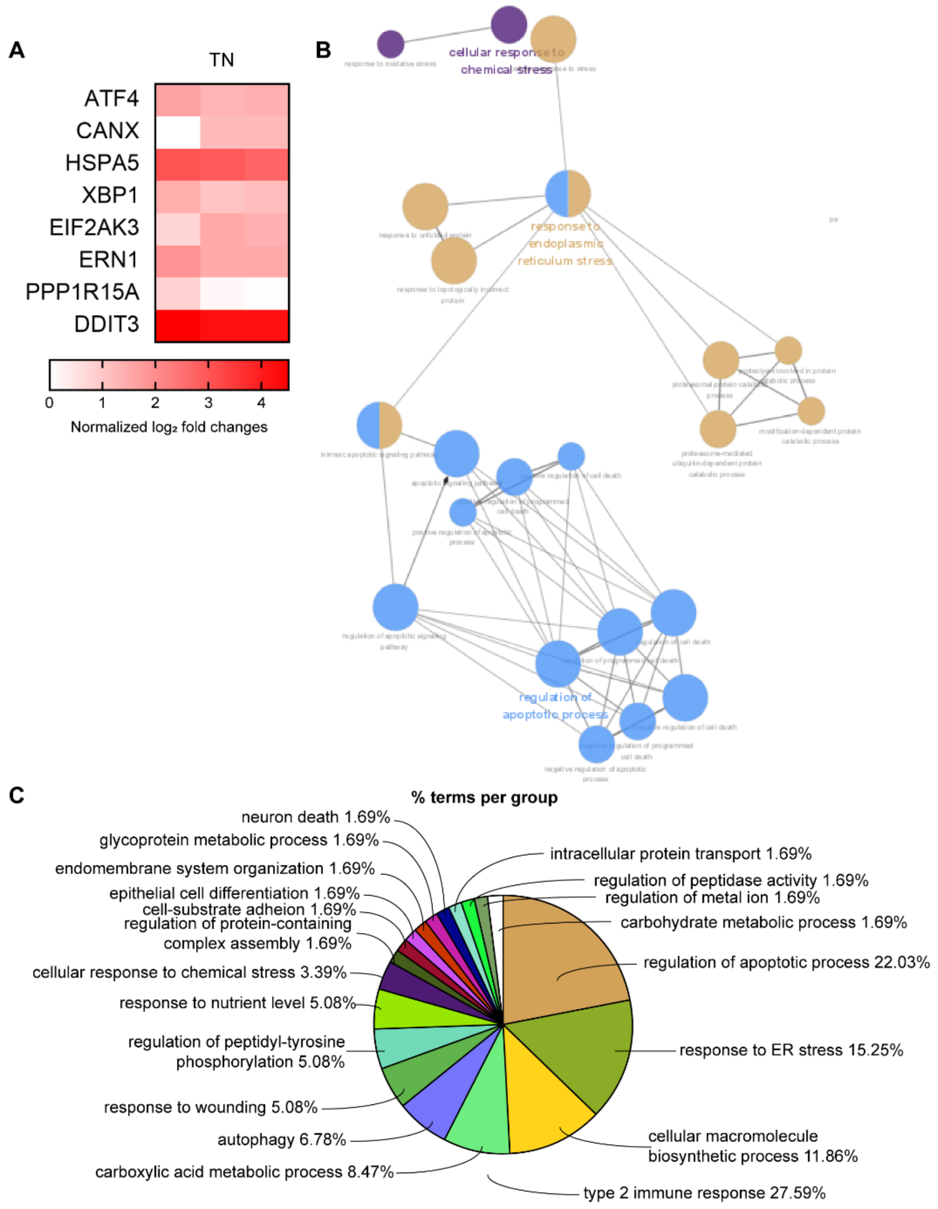

Figure S3. The TN treatment activates apoptotic and ER stress pathways. (A) Heatmap of DEGs related to ER stress upon TN treatment. The color represents the $\log_2$ fold change of genes normalized to DMSO-treated control. Each column represents a $\log_2$ fold change of three biological replicates with TN treatment normalized by the average of control. (B) GO analysis of TN-treated samples. Genes whose $\log_2$ fold change was upregulated over 0.585 and p-value less than 0.05 upon TN treatment were analyzed. (C) A pie chart showing the class of genes whose expression was upregulated by TN treatment.

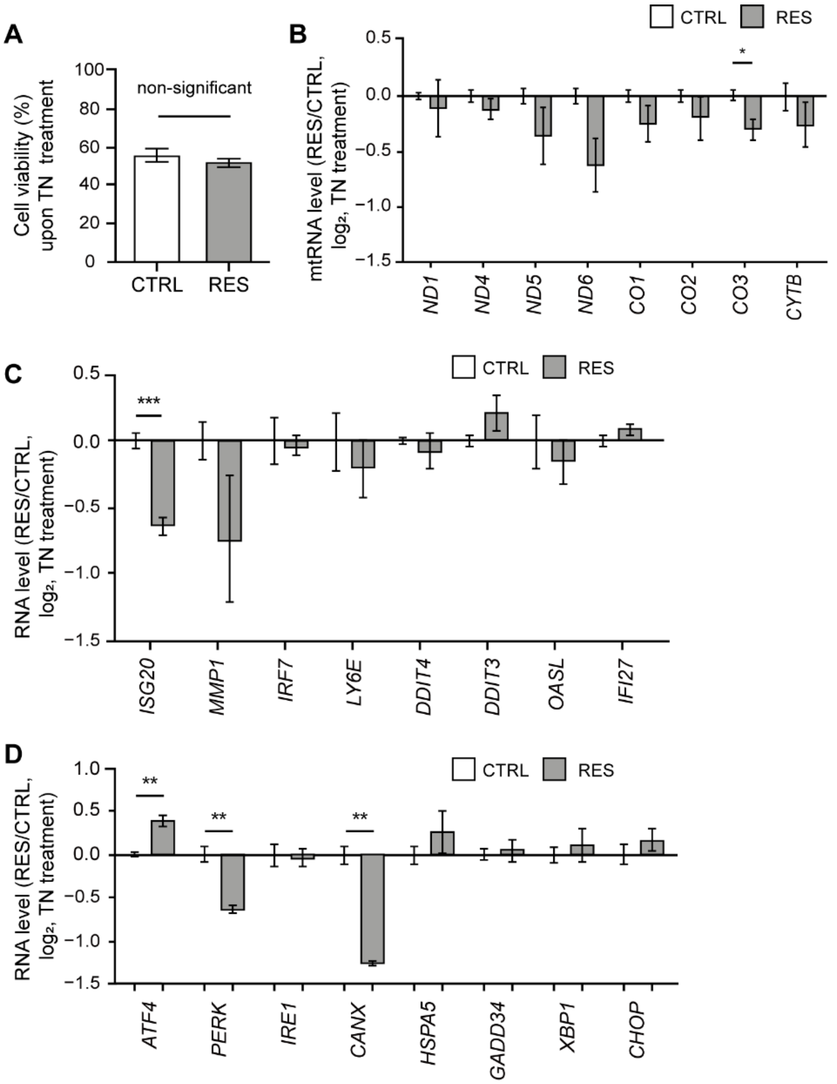

Figure S4. The RES treatment does not rescue ER stress by TN treatment. (A) Cell viability upon TN treatment with or without RES treatment measured by SRB assay. (B-D) The ratios of mtRNAs (B), ISGs (C), and ER stress-related genes (D) in RES and TN co-treated cells compared to those in TN-only treated cells (control). For ratios, Cq values were first normalized to that of *ACTB* mRNA. For untreated samples, the values were normalized to those of control cells without TN treatment. Similarly, RNAs from cells co-treated with RES and TN were normalized separately using RNAs from cells treated with TN only. Unless mentioned otherwise, three independent experiments were carried out, and error bars denote s.e.m. All statistical significances were calculated using one-tailed Student's t-tests, *$p \leq 0.05$, **$p \leq 0.01$, and ***$p \leq 0.001$.

Table S1. Primer sequences for RT-qPCR.

Gene	Forward Primer (5′-3′)	Reverse Primer (5′-3′)
ND1	TCAAACTCAAACTACGCCCTG	GTTGTGATAAGGGTGGAGAGG
ND4	CTCACACTCATTCTCAACCCC	TGTTTGTCGTAGGCAGATGG
ND5	CTAGGCCTTCTTACGAGCC	CGCAAATGGGCGGTAGGCGTGT TTGGGTTGAGGTGATGATG
ND6	TGCTGTGGGTGAAAGAGTATG	CGCAAATGGGCGGTAGGCGTGC CCATAATCATACAAAGCCCC
CO1	GCCATAACCCAATACCAAACG	TTGAGGTTGCGGTCTGTTAG
CO2	CTAGTCCTGTATGCCCTTTTCC	GTAAAGGATGCGTAGGGATGG
CO3	CCTTTTACCACTCCAGCCTAG	CTCCTGATGCGAGTAATACGG
CYTB	CAATTATACCCTAGCCAACCCC	GGATAGTAATAGGGCAAGGACG
IRF7	CTGTGGACACCTGTGACACC	TGCCCTCTCAGGAGCCAA
IFI27	ATCAGCAGTGACCAGTGTGG	ATCAGCAGTGACCAGTGTGG
STAT1	AACCTCGACAGTCTTGGCAC	CACTGAGACATCCTGCCACC
IFIT3	GAAGGAACTGGGCCGCCTGCTAAG	GCCCTGGCCCATTTCCTCACTACC
IκBα	CTCCGAGACTTTCGAGGAAATAC	GCCATTGTAGTTGGTAGCCTTCA
MX1	TTCTGGGTCGGAGGCTACAG	TGGATGGCGGCGTTCT
IRF3	ACACATACTGGGCAGTGAGC	CTACAATGAAGGGCCCCAGG
IFI44	CTGATTACAAAAGAAGACATGACAGAC	AGGCAAAACCAAAGACTCCA
ISG20	GGTGCTGTGCTGTACGACAA	GAGCTGCAGGATCTCTAGCC
MMP1	AGAAAGAAGACAAAGGCAAGTTGA	TTCCCAGTCACTTTCAGCCC
LY6E	CTCCAGGCAGGACGGCCATC	CGAGATTCCCAATGCCGGCACT
OASL	GCGGAGCCCATCACGGTCAC	AGGACCACCGCAGGCCTTGA
DDIT3 (CHOP)	GTTCCAGCCACTCCCCATTA	GTCCCGAAGGAGAAAGGCAA
DDIT4	ACACTTGTGTGCCAACCTGA	CAGGCGCAGTAGTTCTTTGC
ATF4	AGTTCGACTTGGATGCCCTG	CCAACGTGGTCAGAAGGTCA
PERK	TGATGTTGTTTTGGTTGGAGGA	TCGTCACTATCCCATTGGCG
IRE1	CAGCAGACTTTGTCATCGGC	CTCTCGGGTTTTGGTGTCGT
CANX	AGCCAAGAAAGACGATACCGA	GAGATGGCATGATGCTTGGC
HSPA5	CACTCCTGAAGGGGAACGTC	TCAAAGACCGTGTTCTCGGG

GADD34	CTGGCTGGTGGAAGCAGTAA	TATGGGGGATTGCCAGAGGA
XBP1	AAGTTCTGCTTCTGTCGGGG	CTGGGCTGGCACCATGAG
ACTB	CCTGTACGCCAACACAGTGC	ATACTCCTGCTTGCTGATCC
